# Association of Risk Variants in the *CFH* Gene With Elevated Levels of Coagulation and Complement Factors in Idiopathic Multifocal Choroiditis

**DOI:** 10.1001/jamaophthalmol.2023.2557

**Published:** 2023-07-06

**Authors:** Evianne L. de Groot, Jeannette Ossewaarde–van Norel, Joke H. de Boer, Sanne Hiddingh, Bjorn Bakker, Ramon A. C. van Huet, Ninette H. ten Dam–van Loon, Alberta A. H. J. Thiadens, Magda A. Meester-Smoor, Yvonne de Jong–Hesse, Leonoor I. Los, Anneke I. den Hollander, Camiel J. F. Boon, Lambertus A. Kiemeney, Kristel R. van Eijk, Mark K. Bakker, Carel B. Hoyng, Jonas J. W. Kuiper

**Affiliations:** 1 Department of Ophthalmology, University Medical Center Utrecht, Utrecht University, the Netherlands; 2 Center for Translational Immunology, University Medical Center Utrecht, Utrecht University, Utrecht, the Netherlands; 3 Department of Ophthalmology, Donders Institute for Brain, Cognition and Behaviour, Radboud University Medical Center, Nijmegen, the Netherlands; 4 Department of Ophthalmology, Erasmus Medical Centre, Rotterdam, the Netherlands; 5 Department of Ophthalmology, Amsterdam University Medical Centre, Amsterdam, the Netherlands; 6 Department of Ophthalmology, Leiden University Medical Center, Leiden, the Netherlands; 7 Department of Ophthalmology, University Medical Center Groningen, University of Groningen, Groningen, the Netherlands; 8 AbbVie, Genomics Research Center, Cambridge, Massachusetts; 9 Department of Health Evidence, Radboud University Medical Centre, Nijmegen, the Netherlands; 10 Department of Neurology, UMC Utrecht Brain Center, University Medical Center Utrecht, Utrecht University, Utrecht, the Netherlands

## Abstract

**Importance:**

Idiopathic multifocal choroiditis (MFC) is poorly understood, thereby hindering optimal treatment and monitoring of patients.

**Objective:**

To identify the genes and pathways associated with idiopathic MFC.

**Design, Setting, and Participants:**

This was a case-control genome-wide association study (GWAS) and protein study of blood plasma samples conducted from March 2006 to February 2022. This was a multicenter study involving 6 Dutch universities. Participants were grouped into 2 cohorts: cohort 1 consisted of Dutch patients with idiopathic MFC and controls, and cohort 2 consisted of patients with MFC and controls. Plasma samples from patients with idiopathic MFC who had not received treatment were subjected to targeted proteomics. Idiopathic MFC was diagnosed according to the Standardization of Uveitis Nomenclature (SUN) Working Group guidelines for punctate inner choroidopathy and multifocal choroiditis with panuveitis. Data were analyzed from July 2021 to October 2022.

**Main outcomes and measures:**

Genetic variants associated with idiopathic MFC and risk variants associated with plasma protein concentrations in patients.

**Results:**

This study included a total of 4437 participants in cohort 1 (170 [3.8%] Dutch patients with idiopathic MFC and 4267 [96.2%] controls; mean [SD] age, 55 [18] years; 2443 female [55%]) and 1344 participants in cohort 2 (52 [3.9%] patients with MFC and 1292 [96.1%] controls; 737 male [55%]). The primary GWAS association mapped to the *CFH* gene with genome-wide significance (lead variant the A allele of rs7535263; odds ratio [OR], 0.52; 95% CI, 0.41-0.64; *P* = 9.3 × 10^−9^). There was no genome-wide significant association with classical human leukocyte antigen (HLA) alleles (lead classical allele, HLA-A*31:01; *P* = .002). The association with rs7535263 showed consistent direction of effect in an independent cohort of 52 cases and 1292 control samples (combined meta-analysis OR, 0.58; 95% CI, 0.38-0.77; *P* = 3.0 × 10^−8^). In proteomic analysis of 87 patients, the risk allele G of rs7535263 in the *CFH* gene was strongly associated with increased plasma concentrations of factor H–related (FHR) proteins (eg, FHR-2, likelihood ratio test, adjusted *P* = 1.1 × 10^−3^) and proteins involved in platelet activation and the complement cascade.

**Conclusions and relevance:**

Results suggest that *CFH* gene variants increase systemic concentrations of key factors of the complement and coagulation cascades, thereby conferring susceptibility to idiopathic MFC. These findings suggest that the complement and coagulation pathways may be key targets for the treatment of idiopathic MFC.

## Key Points

**Question:**

What genes and pathways are associated with the pathophysiology of idiopathic multifocal choroiditis (MFC)?

**Findings:**

In this case-control genome-wide association study including 170 Dutch cases of idiopathic MFC and 4267 controls in cohort 1 and 52 cases and 1292 controls in cohort 2, an association between the complement factor H (*CFH*) gene and idiopathic MFC was discovered. Complement and coagulation cascade proteins were higher in patients with the risk locus.

**Meaning:**

The results suggest that complement and coagulation cascades play a key role in the underlying disease mechanism of idiopathic MFC.

## Teaser

This case-control study investigates the genes and pathways associated with the pathophysiology of idiopathic multifocal choroiditis.

## Introduction

**Figure 1.**
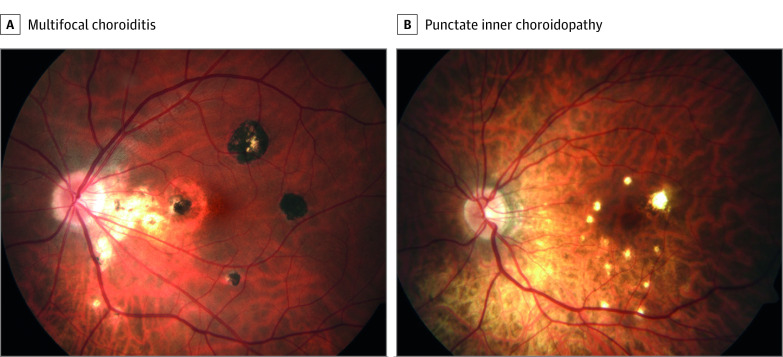
Subtypes of Idiopathic Multifocal Choroiditis Fundus photograph in a patient with multifocal choroiditis (A), and a patient with punctate inner choroidopathy (B).

Idiopathic multifocal choroiditis (MFC) is a rare idiopathic inflammatory choroidopathy in the absence of a systemic disease with an estimated incidence of 1.6 per million population per year. Although more commonly reported among Western European individuals, it is not fully understood how common MFC is among other ancestral groups. Idiopathic MFC is characterized by inflammation of the choriocapillaris, loss of retinal pigment epithelium, outer retinal ischemia, and the frequent development of choroidal neovascularization.^1,2,3^ Patients with idiopathic MFC are predominantly young women with myopia who appear otherwise healthy, who experience vision loss, metamorphopsia, scotomas, and photopsias.^2,4^ Idiopathic MFC may manifest with or without panuveitis, and there is a variable degree of involvement of the peripheral retina. Idiopathic MFC without involvement of the peripheral retina and without cells in the anterior or vitreous cavity is often referred to as punctate inner choroidopathy (PIC) (Figure 1).^4,5,6,7^ However, definite standardization of disease definitions is lacking, and whether MFC and PIC are indeed separate disease entities remains a subject of debate.^5,8,9,10^

Molecular profiling studies of idiopathic MFC remain sparse. Previous studies revealed changes in platelet granularity,^11^ whereas candidate gene association studies have linked complement and interleukin encoding genes to idiopathic MFC.^12,13^ This supports an inflammatory basis for this disease in combination with coagulation dysfunction. This is also evident from the efficacy of immunosuppressive agents—including disease-modifying antirheumatic drugs (DMARDs)—used as treatment for idiopathic MFC.^14,15,16^ However, how these factors contribute to the disease remains uncertain. Better understanding of the key disease pathways driving idiopathic MFC and a molecular basis in support of the clinical categories are needed to improve disease management. To allow identification of genetic variants that confer susceptibility to idiopathic MFC, we conducted a genome-wide association study (GWAS) and targeted proteomic profiling in a Dutch cohort.

## Methods

### Study Population

This study was performed following the tenets of the Declaration of Helsinki and its further amendments. Institutional review board approval was obtained from all participating centers. Patients were recruited from the following centers in the Netherlands: University Medical Center Utrecht, Radboud University Medical Center, Amsterdam University Medical Center, Leiden University Medical Center, Erasmus Medical Center, and University Medical Center Groningen. All patients provided written informed consent and did not receive a stipend or other form of compensation for participating. This study followed the Strengthening the Reporting of Observational Studies in Epidemiology (STROBE) reporting guidelines.

Each case was evaluated according to the Standardization of Uveitis Nomenclature (SUN) Working Group guidelines,^8,9^ and evaluation included standard diagnostic workup for uveitis, comprising routine laboratory testing, chest radiography, and when indicated, positron emission tomography–computed tomography, anterior chamber paracentesis with polymerase chain reaction and/or Goldmann-Witmer index, and cytokine analysis. The diagnosis idiopathic MFC was established for patients who presented with chorioretinal lesions in the posterior pole in the absence of papillitis and retinal vasculitis and without evidence of an ocular infection, sarcoidosis, tuberculosis, inflammatory bowel disease, birdshot chorioretinopathy, or vitreoretinal lymphoma. Patients with idiopathic MFC were subdivided into 2 subtypes: MFC and PIC. Subtype classification was in accordance with the SUN Working Group with adjustments based on consensus by an expert panel (ie, uveitis and/or retina specialists of participating centers) (Figure 1).^8,9^ Patients were diagnosed with PIC if (1) chorioretinal lesions were located within the posterior pole with or without involvement of the midperipheral retina but without involvement of the peripheral retina, (2) no cells were observed in the anterior chamber or vitreous (grade 0), and (3) predominant lesion size was less than 250 μm with a punctate appearance. MFC diagnosis was established if (1) chorioretinal lesions were located both in the posterior pole and extended to the peripheral retina, and/or (2) cells were observed in the anterior chamber or vitreous (>1+ cells), and/or (3) predominant lesion size was greater than 125 μm with a round appearance. The MFC/PIC group included cases with insufficient clinical data to distinguish MFC from PIC. Race and ethnicity data were not gathered for the participants of this study because these data were not standardized at the participating centers.

### Single-Nucleotide Variation Array Genotyping

Details on the description of the genomic DNA extraction are shown in the eMethods in Supplement 1. Patients in cohort 1 were genotyped with the Infinium OmniExpress-24 bead chip (Illumina) at the Genome Analysis Center, Helmholtz Zentrum, in München, Germany. Data from Dutch population controls from the Nijmegen Biomedical Study who were genotyped with either the Infinium OmniExpress-12 bead chip (Illumina) or the Infinium OmniExpress-24 bead chip were used as reference for cohort 1.^17^ We genotyped the rs7535263 variant in an independent Dutch cohort (cohort 2) using TaqMan single-nucleotide variation (SNV) genotyping technology (Thermo Fisher Scientific) and compared these with genotype data from Dutch control participants from an amyotrophic lateral sclerosis study.^18^ The genotyping results from both cohorts were combined for meta-analysis.

### Targeted Proteomics of Blood Plasma

Available plasma samples from patients in cohort 1 with idiopathic MFC who had not received treatment at the time of the blood withdrawal were centrifuged at 2000*g* for 10 minutes at room temperature and stored directly at −80 °C. Samples from patients were thawed and randomized over plates before the samples were shipped on dry ice to the protein biomarker platform Olink Proteomics (Erasmus Medical Center, Rotterdam, the Netherlands). The Explore 384 Inflammation II biomarker panel (Olink) was used to measure 370 proteins associated with inflammatory responses. The protein biomarker platform uses proximity extension assay technology to measure protein concentrations by next-generation sequencing. The protein expression data are expressed in arbitrary units (normalized protein expression) and represent the relative protein concentration in the plasma of the sample on a log2 scale.^19,20^ The full list of measured proteins in the Inflammation II panel is in eTable 6 in Supplement 2. The association between the genotype of the lead variant rs7535263 and plasma protein concentrations was assessed using a likelihood ratio test adjusted for age and sex. We performed functional enrichment analysis with the genotype associated plasma proteins (*P* < .05) to determine which pathways these proteins belong to using the clusterProfiler R package (R Project for Statistical Computing).^21^ The Reactome database was used as a reference in enrichment analysis. Results were visualized using the dotplot function.

### Statistical Analysis

A detailed description of the quality controls and imputation steps are listed in the eMethods and eFigures 1 to 3 in Supplement 1. The single-variant association tests were performed with the Scalable and Accurate Implementation of Generalized mixed model (SAIGE), version 0.29.3, and were performed in R, version 4.2.8 (R Project for Statistical Computing).^22^ To determine the appropriate number of principal components (PCs) to correct for population stratification, we performed a general linear model with the first 10 PCs, sex, and phenotype, and identified the largest PC that was significantly associated (2-sided *P* < .05) with the phenotype and selected all PCs up to that last significant PC. This resulted in a final model with the covariates age, sex, and the first 6 PCs. Association testing was performed for all cases in cohort 1 and for the subtypes MFC and PIC separately. Study data were analyzed July 2021 to October 2022.

## Results

### Variants at 1q31 and Idiopathic MFC

**Figure 2.**
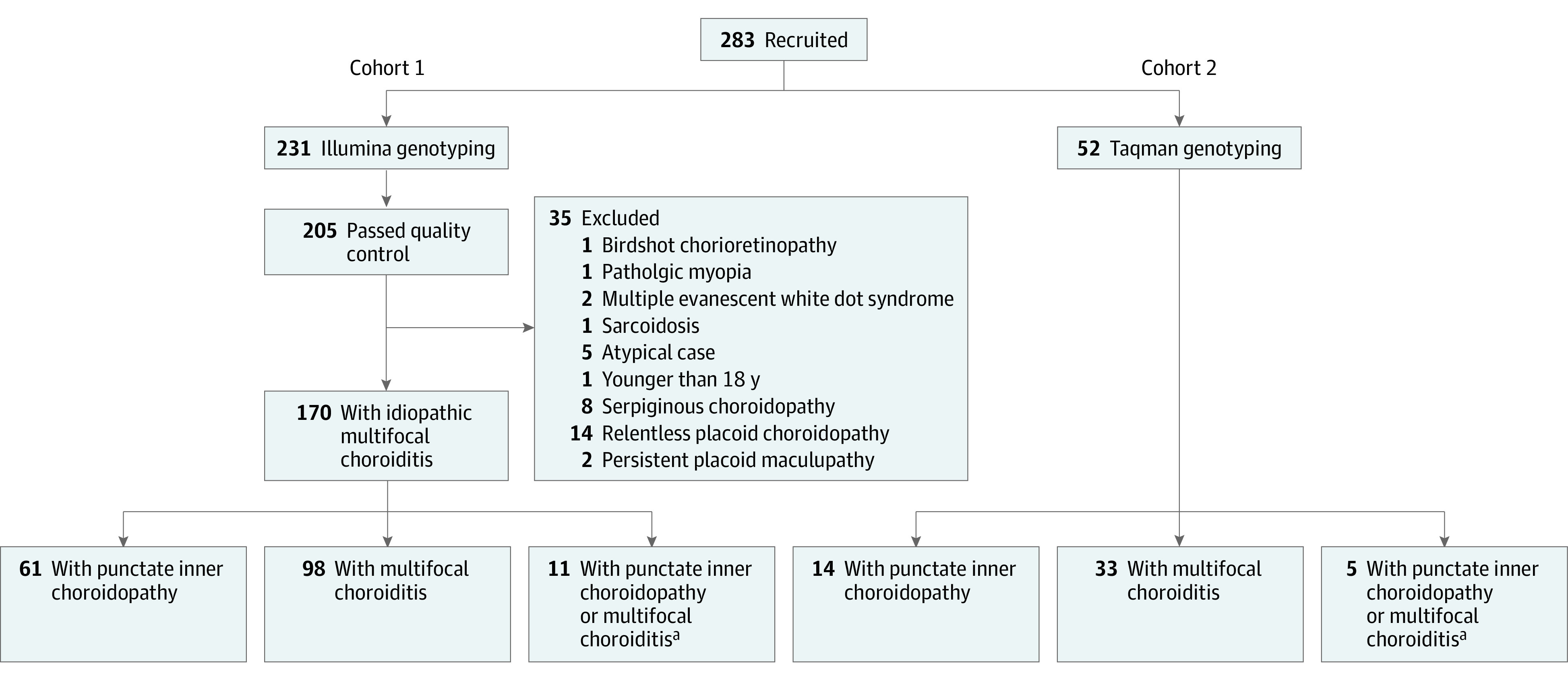
Overview of All Recruited Patients in Cohorts 1 and 2 For cohort 1, after quality control and case selection, 170 idiopathic cases of multifocal choroiditis were analyzed. ^a^Insufficient data for accurate subtype classification between multifocal choroiditis or punctate inner choroidopathy.

For cohort 1, a total of 231 patients were recruited and from them, genomic DNA was isolated from peripheral blood cells. In total, 35 cases were removed after quality control of genotyping because after inclusion, the patients were diagnosed with an alternative diagnosis (Figure 2). After quality control and imputation, 205 MFC cases and 4267 controls were included in analysis, and 8 653 120 SNVs were tested. For replication, we recruited an independent Dutch cohort of 52 patient cases with idiopathic MFC (cohort 2) (Figure 2).

**Figure 3.**
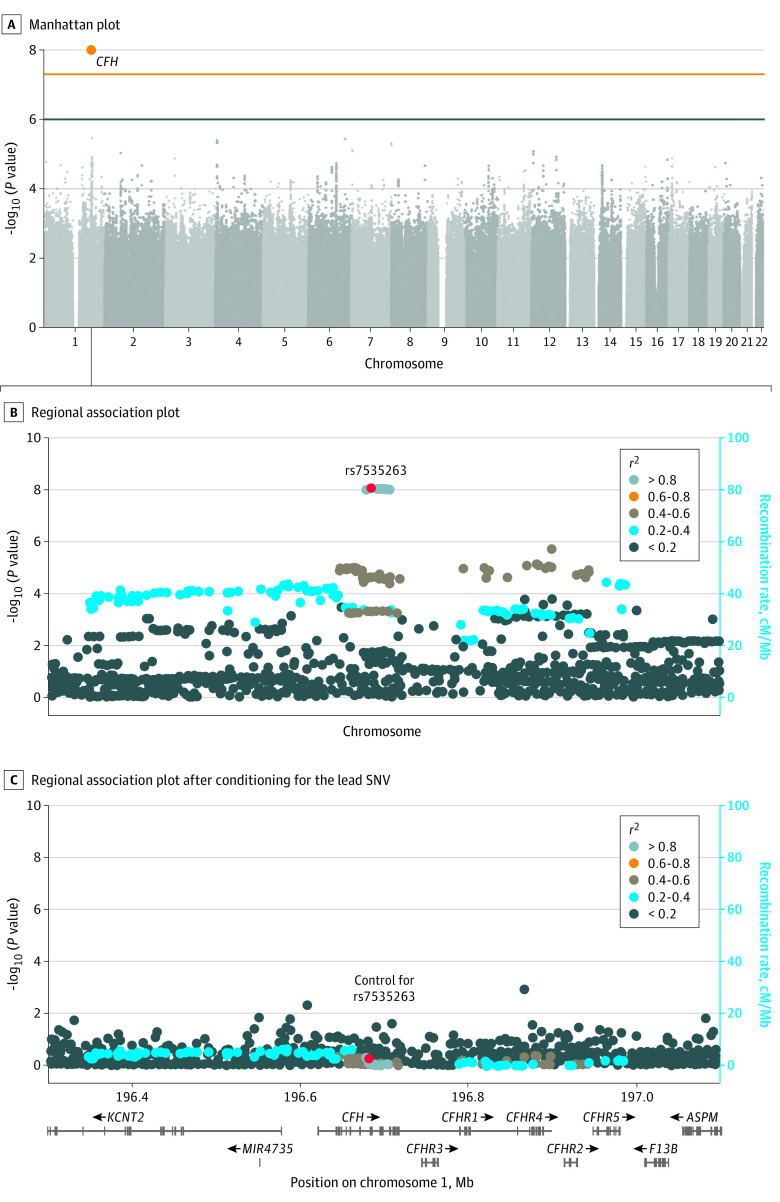
Manhattan and Regional Plot of Genome-Wide Association Analysis of Idiopathic Multifocal Choroiditis or Punctate Inner Choroidopathy A, Manhattan plot of the results of a genome-wide association study in 170 patients with idiopathic multifocal choroiditis or punctate inner choroidopathy and 4266 Dutch control participants. Each dot represents the result of the association test for a single-nucleotide variation (SNV). On the x-axis, the chromosomes are displayed, and the y-axis represents the −log_10_ of the *P* value from a scalable and accurate implementation of generalized mixed model including the covariates age, sex, and the first 6 principal components from ancestry analysis. The blue line indicates the threshold for suggestive association (*P* < 1.0 × 10^−6^), the orange line indicates the threshold for genome-wide significance (*P* < 5 × 10^−8^). One genome-wide significant signal was observed in the *CFH* gene (20 SNVs; lead variant the A allele of rs7535263). Regional association plot generated in LocusZoom of the *CFH* region before (B) and after (C) conditioning on rs7535263. On the x-axis, the position on the chromosome is displayed including the position of the genes in this region. The left y-axis represents the −log_10_(*P *value) of the results of the association tests, and the right y-axis represents the recombination rate. The lead SNV is plotted as a red dot; other data points are colored according to their *r*^2^ with the lead SNV.

**Figure 4.**
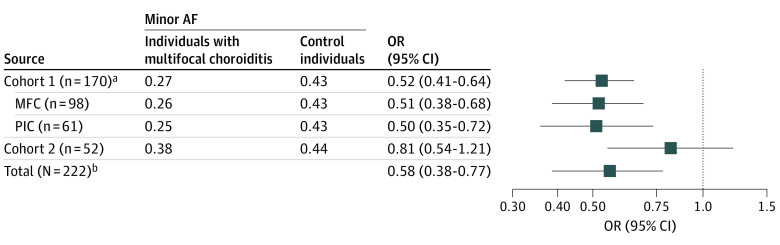
Forest Plot Depicting the Summary Statistics of the Association Analysis for Different Disease Groups for the A Allele of rs7535263 in the *CFH* Gene Controls for cohort 1 include the 4266 population control participants used in genome-wide association analysis, and controls for cohort 2 include 1292 population control participants. AF indicates allele frequency; MFC, multifocal choroiditis; OR, odds ratio; PIC, punctate inner choroidopathy. ^a^Insufficient data for accurate subtype classification between MFC or PIC for 11 cases. ^b^Results of a meta-analysis of cohort 1 (n = 170) and cohort 2 (n = 52).

To identify idiopathic MFC susceptibility genes, we conducted genome-wide and major histocompatibility complex (MHC) region association analysis for cases in cohort 1. After quality control, imputation, and exclusion of patients with alternative diagnoses, this study included a total of 4437 participants in cohort 1 (170 [3.8%] Dutch patients with idiopathic MFC and 4267 [96.2%] controls; mean [SD] age, 55 [18] years; 1993 male [45%]; 2243 female [55%]) and 1344 participants in cohort 2 (52 [3.9%] cases and 1292 [96.1%] controls; 737 male [55%]; 607 female [45%]) (Figure 2). The clinical characteristics for cases and controls are shown in eTables 1 and 2 in Supplement 1. We used an additive logistic mixed model, adjusted for age, sex, and the top 6 PCs from ancestry analysis for genome-wide association testing. We identified a genome-wide significant association on chromosome 1 with the A allele of rs7535263 in the complement factor H (*CFH*) gene (odds ratio [OR], 0.52; 95% CI, 0.41-0.64; *P* = 9.3 × 10^−9^) (Figure 3 and Figure 4). Both subtypes PIC and MFC demonstrated nearly identical allele frequency in cases (Figure 4). The risk allele, G, of rs7535263 had an allele frequency of 73% (248 of 340) in cases compared with 57% (3670 of 8534) in controls, of which the latter is similar to the European superpopulation of the 1000 Genomes Project (580 of 1006 [58%]).

Due to strong linkage disequilibrium (LD), in total, 20 SNVs across the *CFH* locus showed genome-wide significant association (*P* < 5.0 × 10^−8^) (eTable 3 in Supplement 2). After conditioning for the SNV rs7535263 in *CFH*, the association signal of the remaining variants in *CFH* was mitigated (Figure 3). The risk allele G of rs7535263 was also more frequent in cases (allele frequency, 64 of 104 [62%]) of cohort 2 compared with controls (allele frequency, 1451 of 2584 [56%]) but did not reach statistical significance, most likely representing an underpowered comparison (combined meta-analysis (OR, 0.58; 95% CI, 0.38-0.77; *P* = 3.0 × 10^−8^) (Figure 4).

We also imputed and tested gene variants and classical alleles in the MHC region in cohort 1, but no genome-wide association in the MHC region was detected (lead variant in intron 5 of *HLA-DRB1*, *P* = 3.7 × 10^−4^) nor was any classical HLA allele markedly associated (lead classic HLA allele, HLA-A*31; *P* = .002) (eTables 4 and 5 in Supplement 2 and eFigure 4 in Supplement 1).

### Idiopathic MFC Risk Variant, Plasma Coagulation, and Complement Protein Levels

**Figure 5.**
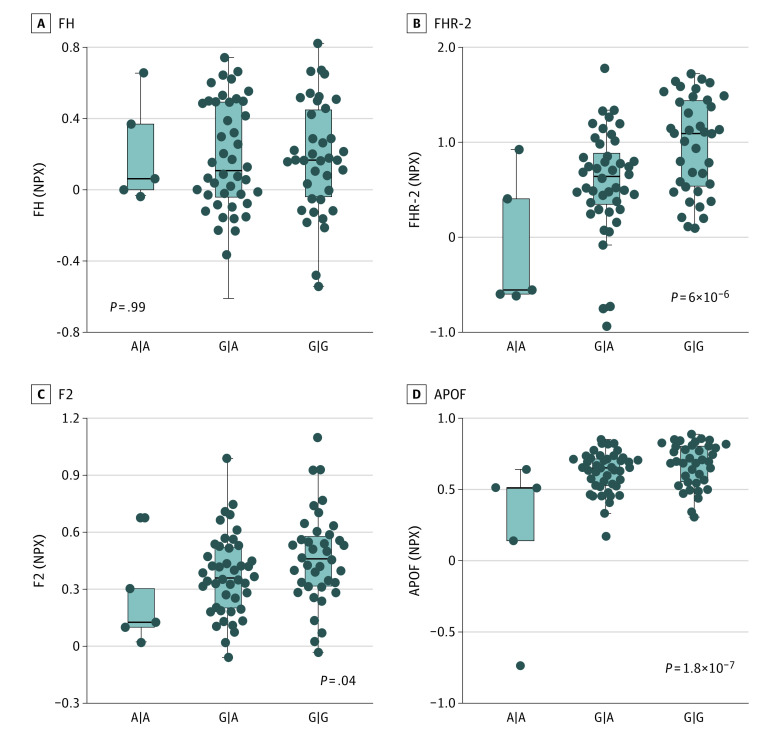
Association Between the rs7535263 in *CFH* and Plasma Concentrations of Immune Mediators in Multifocal Choroiditis EDTA plasma was subjected to targeted 370-plex proteomics array (Olink) in 87 treatment-naive patients with idiopathic multifocal choroiditis or punctate inner choroidopathy. This figure shows the plasma levels for complement factor H (FH) (A), complement factor H–related protein 2 (FHR-2) (B), prothrombin (F2) (C), and apolipoprotein F (APOF) (D) across the genotype of rs7535263 in patients. Protein concentrations are expressed as normalized protein expression (NPX).

The *CFH* gene and *CFH*-related genes (*CFHR1-5*) cluster together on chromosome *1q31*. The variants (in LD with rs7535263) at *1q31* identified in this study were recently shown to be associated with circulating factor H–related (FHR) protein concentrations.^23,24^ To investigate the association between the genetic signal at *1q31* and the plasma proteome, we used a 370-plex proximity extension assay for targeted proteomics of 87 treatment-free patients from this study. We performed an association analysis of the top idiopathic MFC variant rs7535263 in the *CFH* gene with plasma protein concentrations. Adjusting for sex and age, the risk allele G of rs7535263 was associated with differential plasma levels of 27 proteins (Figure 5; eFigure 5 in Supplement 1), including FHR protein 2, FHR-4, FHR-5, complement C5, coagulation factors such as prothrombin (F2) and fibrinogen alpha chain, and in proteins involved in lipid metabolism (eg, apolipoprotein F [APOF], apolipoprotein C-I, and gastric inhibitory polypeptide receptor) (eTable 6 in Supplement 2). No association with FH protein (encoded by the *CFH* gene) concentrations were observed with rs7535263 (Figure 5). However, associations were found with FHR-2 (adjusted *P = *1.1 × 10^−3^) and APOF (adjusted *P* = 6.6 × 10^−5^), which were increased in plasma of cases carrying the risk allele G of rs7535263 (Figure 5). Pathway enrichment analysis of the 27 proteins associated with the rs7535263 genotype revealed strong enrichment for complement (eg, regulation of complement cascade R-HSA-977606; adjusted *P* = 1.1 × 10^−7^) and coagulation cascades (eg, platelet activation, signaling, and aggregation R-HSA-76002; adjusted *P* = 4.4 × 10^−5^) (eFigure 6 in Supplement 1). Considered collectively, we identified rs7535263 in the *CFH* locus as a novel risk variant for idiopathic MFC that was associated with increased circulating levels of key factors of complement and coagulation cascades.

## Discussion

We identified genetic variants at *1q31* at the extended *CFH* locus that were associated with idiopathic MFC with nearly identical allele frequencies for the subtypes MFC and PIC. Our findings further suggest that patients who carry the risk alleles exhibited increased plasma levels of *CFH*-associated proteins and other proteins associated with the complement cascade and platelet degranulation pathways. This supports the hypothesis that coagulation and inflammatory processes may contribute to the disease biology of idiopathic MFC.

Recently, classification criteria for MFC with panuveitis and PIC have been proposed. These include differentiating criteria for PIC and MFC, such as the location and size of the chorioretinal lesions and the presence of inflammation in the vitreous or anterior chamber.^8,9^ The close clinical similarities of MFC and PIC subtypes suggest that they may actually represent the same disease entity with MFC representing cases with more profound inflammation.^25,26^ Using an approach with idiopathic MFC clinical phenotypes only distinguished by differential peripheral retinal involvement, lesion size, and the presence of intraocular inflammation revealed that both phenotypically related subtypes share a genetic association with the *CFH* locus on chromosome *1q31* with almost identical allele frequency of the lead variant in both subtypes. Our results suggest that both subtypes have a similar molecular basis. However, comprehensive assessment of the genetic correlation between PIC and MFC requires a much larger sample size in subsequent GWASs.

There are few genetic studies in MFC.^12,13^ The *IL10* and *TNF* genes have been linked to MFC in other studies, but association with these SNVs did not reach genome-wide signals in our study.^12^ This study identified a variant in the *CFH* locus that associates with idiopathic MFC. This risk variant is in full LD with rs10922109, a variant that is associated with age-related macular degeneration (AMD)^23,24,27^ and rs1410996 that has previously been associated with MFC using targeted SNV analysis carried out on 48 cases.^13^ Among the established genetic loci associated with AMD,^28^ we detected only a suggestive association for another variant in the *CFH* gene (rs570618 LD = 0.99 with rs1061170[Y402H]; *P* = 1.1 × 10^−5^) (eTable 7 in Supplement 2). On the contrary, although being a risk factor for AMD and idiopathic MFC, the G allele of rs7535263 is protective for central serous chorioretinopathy, another disease affecting the retinal pigment epithelium and choroid.^29,30^ Interestingly, patients with all these conditions are prone to develop choroidal neovascularization. The shared association with a locus in the *CFH* gene suggests that choroid/retinal pigment epithelium homeostasis is strongly influenced by genetic variation at *1q31*. Even though 75% of the affected eyes in the current study were myopic, we did not find an association with risk loci observed in myopia.^31,32,33,34,35^ However, it is possible this is due to insufficient power to detect associations with these risk loci.

Using plasma proteomics of treatment-free patients with idiopathic MFC, we demonstrated that the genotype of rs7535263 in *CFH* was not associated with the levels of its encoding protein FH but instead was strongly associated with increased plasma concentration of FHR proteins, such as FHR-2, FHR-4, and FHR-5. These findings are in line with recent studies in patients with AMD that also showed that the G allele of rs7535263 (and variants in strong LD with it) is associated with increased FHR-2, FHR-4, and FHR-5 levels.^23,24,36,37^ FHR-2, FHR-4, and FHR-5 have been shown to accumulate in the intercapillary septa of the choriocapillaris in AMD and to interrupt regulation of the complement cascade by competing with FH-binding sites.^23^ These observations are of interest for the understanding of the disease biology of idiopathic MFC. Potentially, the genetic predisposition to higher systemic levels of FHRs in idiopathic MFC may result in the accumulation of FHR proteins in the choriocapillaris and subsequently, to local increased complement activation.

Our proteomic analysis revealed that the *CFH* risk variant also associates with increased levels of proteins involved in lipid metabolism (eg, apolipoproteins) and key components of the coagulation pathway (eg, prothrombin). The association of risk variants in *CFH* with circulating coagulation factors has also previously been reported in a plasma proteomic analysis in 4998 healthy blood donors, indicating that the here-described genetic predisposition in the extended *CFH* locus with altered levels of coagulation pathway factors is robust.^38^ The association between variants in *CFH* and coagulation pathway factors, such as prothrombin (F2), is not yet reported in large proteomic studies of AMD. An explanation could be that these studies measured protein concentrations in serum, which could influence quantification of coagulation factors.

The association of the *CFH* risk locus with plasma coagulation factors is of interest because there is increasing evidence for a cross-talk between coagulation factors and the complement cascade.^39,40^ In idiopathic MFC, hypoperfusion of the choriocapillaris is observed on imaging modalities, which is hypothesized to be the result of microthrombi in the choriocapillaris.^11^ Elevated plasma FHR proteins have been linked to prothrombotic events,^41^ whereas genetic disruption of FH-binding leads to complement component 3 (C3)–mediated ischemic retinopathy and cotton wool spots in mice.^42^ Because FH and FHR-2, FHR-4, and FHR-5 compete for C3,^23,43^ it is tempting to speculate that elevated systemic levels of FHR-2, FHR-4, or FHR-5 may contribute to C3 accumulation and ischemia in the choriocapillaris in idiopathic MFC. Treatment with immunomodulatory therapy including DMARDs and biologicals positively influences the disease course in idiopathic MFC.^14,15,16,44^ Interestingly, we previously reported that patients with idiopathic MFC who demonstrate increased platelet granularity characteristics in blood often require immunomodulatory therapy to control the disease activity.^11^

Taken together, we hypothesize that an increased activity of the complement system and coagulation cascade leads to a local inflammatory response and the development of microthrombi/ischemia in the choriocapillaris. This supports the use of immunomodulatory treatment (DMARD/biologicals) in these patients. Given the shared genetic predisposition at the extended *CFH* locus with AMD, it is tempting to speculate that emerging treatments that target *CFH* downstream C3 and C5 for treatment of AMD may be repurposed for management of idiopathic MFC.^45,46^ However, future research should determine the involvement of C3 and C5 in idiopathic MFC disease mechanisms.

### Strengths and Limitations

To our knowledge, this study included the largest-reported genetic analysis of idiopathic MFC thus far. However, owing to the rarity of the condition, we performed a GWAS in a relatively small number of cases. This influenced the power and limited the ability to detect association to variants with relatively large effect size and hampered efforts to replicate statistically the signal in the *CFH* gene in an independent cohort. However, the *CFH* risk locus demonstrated consistent direction of effect in cohort 2, and we demonstrated functional implications of the GWAS signal on plasma protein concentrations in patients with idiopathic MFC. Patients with MFC are more likely to have myopia. Ophthalmological data (eg, the axial length) were not available for the control participants, but it would be interesting to further study the interaction of clinical data with risk variants in *CFH* in larger-powered studies.

## Conclusions

In conclusion, findings of this case-control study suggest that a locus in the *CFH* gene was associated with the development of idiopathic MFC in individuals, and this locus was also associated with the concentration of circulating proteins with key functions in coagulation and complement cascades. This may suggest a key role of the complement and coagulation cascades in the underlying disease mechanism of idiopathic MFC.

## Notes

Supplement 1.
eMethods
**eTable 1.** Clinical Characteristics of Cases in Cohort 1 and 2 **eTable 2.** Clinical Characteristics of All Subjects Included in Genome-Wide Association **eFigure 1.** Principal Component Analysis **eFigure 2.** Quantile-Quantile Plot **eFigure 3.** Overview of Quality Control Steps **eFigure 4.** Association Results of the MHC Region **eFigure 5.** Heatmap Correlation **eFigure 6.** Reactome Enrichment Analysis

Supplement 2. **eTable 3.** Genome-Wide Significant Hits in Cohort 1 **eTable 4.** Association Results for Classical HLA Alleles With 4-Digit Imputation **eTable 5.** Association Results for Classical HLA Alleles With G Group Imputation **eTable 6.** Full List of Proteins Measured in the Olink Explore 384 Inflammation II Panel **eTable 7.** Summary Statistics of the 52 Risk Loci for Age-Related Macular Degeneration

Supplement 3. Data Sharing Statement
