# Supplementary material for: Association of Risk Variants in the CFH Gene With Elevated Levels of Coagulation and Complement Factors in Idiopathic Multifocal Choroiditis
Source: JAMA Ophthalmol. 2023 Jul 6;141(8):737–45. doi: 10.1001/jamaophthalmol.2023.2557 (PMC10326733; doi:10.1001/jamaophthalmol.2023.2557)
Supplement: Supplement 1. — eMethods eTable 1. Clinical Characteristics of Cases in Cohort 1 and 2 eTable 2. Clinical Characteristics of All Subjects Included in Genome-Wide Association eFigure 1. Principal Component Analysis eFigure 2. Quantile-Quantile Plot eFigure 3. Overview of Quality Control Steps eFigure 4. Association Results of the MHC Region eFigure 5. Heatmap Correlation eFigure 6. Reactome Enrichment Analysis [file jamaophthalmol-e232557-s001.pdf]

Supplementary Online Content

de Groot EL, Ossewaarde–van Norel J, de Boer JH, et al. Association of risk variants in the *CFH* gene with elevated levels of coagulation and complement factors in idiopathic multifocal choroiditis. *JAMA Ophthalmol*. Published online July 6, 2023. doi:10.1001/jamaophthalmol.2023.2557

eMethods

eTable 1. Clinical Characteristics of Cases in Cohort 1 and 2

eTable 2. Clinical Characteristics of All Subjects Included in Genome-Wide Association

eFigure 1. Principal Component Analysis

eFigure 2. Quantile-Quantile Plot

eFigure 3. Overview of Quality Control Steps

eFigure 4. Association Results of the MHC Region

eFigure 5. Heatmap Correlation

eFigure 6. Reactome Enrichment Analysis

This supplementary material has been provided by the authors to give readers additional information about their work.

© 2023 de Groot EL et al. *JAMA Ophthalmology.*

eMethods

Genomic DNA extraction

The samples for genetic analysis were collected in two cohorts. Cohort 1 (n=231) consisted of samples obtained between 2006 and 2021 in the EMC, UMCU, Radboudumc and LUMC. Cohort 2 (n=52) consisted of samples obtained between 2021 and 2022 in the Radboudumc, UMCU, AUMC and UMCG. For cohort 1, genomic DNA isolation was performed at the Radboudumc, UMCU and the EMC. In the UMCU and Radboudumc DNA isolation was performed using the Hamilton Microlab STAR autoload system with an integrated Chemagen MSM I separation module (Hamilton Robotics GmbH, Martinsried, Germany) and the DNA was isolated with the Chemagic DNA blood kit special (PerkinElmer). In the EMC the DNA isolation was performed with the Tecan Freedom EVO®-HSM Workstation using the ReliaPrep™ Large Volume HT gDNA Isolation System chemistry. Following DNA isolation, all samples were sent to the Radboudumc where the concentrations of DNA fractions were determined (Hamilton Microlab Starlet robot with integrated Tecan Infinite 200 Pro reader) and the samples were checked for degradation by 1% agarose gel electrophoresis before the samples were genotyped. For cohort 2, genomic DNA isolation was performed either with the Hamilton Microlab STAR or manually with the AllPrep DNA/RNA/miRNA Universal Kit (Qiagen, Hilden, Germany) following standard protocol.

Quality control and Imputation

Quality control (QC) was performed in PLINK (v1.90b3z 64-bit) for samples (i.e., sample call rate, sex mis-match, population outliers and duplicates/relatedness) and single nucleotide polymorphisms (SNPs) (SNP call rate, multiallelic SNPs, minor allele frequency (MAF), deviation from the Hardy-Weinberg equilibrium (HWE). Ancestry was confirmed with principal components analysis (PCA) in PLINK using the third phase of the International HapMap project (Hapmap3) data as anchoring populations. As shown in **eFigure 1**, most cases and controls were from Western European ancestry (identical to CEU - Northern and Western European ancestry population). We removed cases and controls >4 standard deviations from the CEU population. After QC, samples from 205 cases and 4267 control subjects were used for analysis. Data were phased using Eagle 2.4 and were imputed using the HRC reference panel (1.1) on the Michigan Imputation Server using the default threshold INFO score >0.3.[1] All monomorphic and multi-allelic SNPs were removed. After imputation, SNPs with a calculated INFO scores (Rsquare) <0.6 were removed followed by post-imputation QC (**eFigure 3**). After imputation 8,653,120 SNPs passed the quality control check and were used for analysis (**eFigure 2**).

The Major histocompatibility complex (MHC) region was imputed with the Michigan Imputation Server pipeline using a MHC-specific panel including the default script for Imputation preparation and checking provided by the Michigan server (v4.2.7) using the Haplotype Reference Consortium v1.1. This pipeline results in the imputation of classical Human Leukocyte Antigen (HLA) alleles, amino acid residues, SNPs, and insertions/deletions in the *MHC* region. The imputation of classical HLA alleles provides accurate estimates of HLA types at two-digit resolution (e.g. HLA-A*29 'serotypes') and four-digit resolution (e.g. HLA-*A29:02, i.e., unique amino acid sequence of the gene product). G-group resolution determines the sequences of the exons encoding the peptide binding groove. G-group alleles can map to multiple two-field HLA alleles.[2]

1. Das S, Forer L, Schönherr S, et al. Next-generation genotype imputation service and methods. *Nat Genet.* 2016;48(10):1284-1287. doi:10.1038/ng.3656
2. Luo Y, Kanai M, Choi W, et al. A high-resolution HLA reference panel capturing global population diversity enables multi-ancestry fine-mapping in HIV host response. *Nat Genet.* 2021;53(10):1504-1516. doi:10.1038/s41588-021-00935-7

© 2023 de Groot EL et al. *JAMA Ophthalmology.*

eTable 1. Clinical characteristics of cases in cohort 1 and 2

	**Cohort 1**<br>n=170 patients<br>n=250 eyes	**Cohort 2**<br>n=52 patients<br>n=86 eyes
Female gender, n(%) of all patients	158 (93)	42 (81)
Mean (SD) age of all patients	43 (13)	45 (13)
Subtype, n (%) of all patients		
PIC	61 (36)	14 (27)
MFC	98 (58)	33 (63)
MFC/PIC	11 (6)	5 (10)
Bilateral disease, n (%) of all patients	80 (47)	34 (65)
History of CNV, n (%) of all patients	126 (74)	36 (69)
Median LogMAR BCVA [IQR] of all affected eyes	0.09 [-0.04 – 0.40]	0.03 [0.07 – 0.41]
Median myopia [IQR] of all affected eyes	-4.5 [-7.75 - -0.75]	-3.0 [-7.6 – 0.0]

MFC, multifocal choroiditis; PIC, punctate inner choroidopathy; CNV, choroidal neovascularization; LogMAR, Logarithm of the Minimum Angle of Resolution; BCVA, best-corrected visual acuity. Missing data consisted of: bilateral disease 1 patient, history of CNV 2 patients, BCVA 2 eyes, myopia 19 eyes

eTable 2. Clinical characteristics of all subjects included in genome-wide association analysis

	**Cases cohort 1**<br>n=170	**Controls cohort 1**<br>N=4267
Mean (SD) age, years	43 (13)	55 (18)
Female gender, n (%)	158 (93)	2285 (54)

© 2023 de Groot EL et al. *JAMA Ophthalmology.*

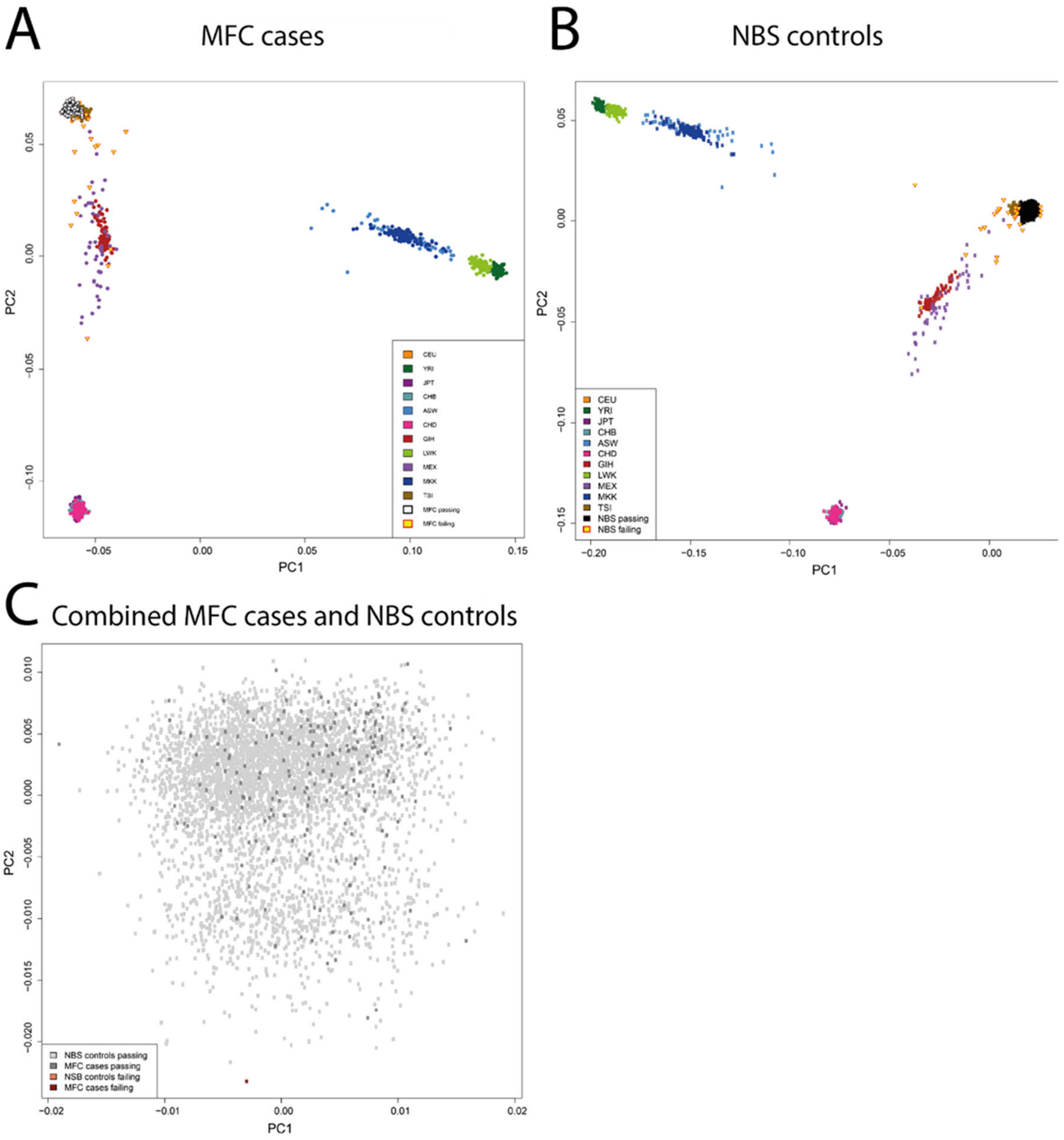

eFigure 1. Principal component analysis. The visualization of the first 2 principal components in cases (**A**), NBS controls (**B**) and the combined cohort (**C**). The colors in A and B indicate the Hapmap3 samples as stated in the legend and the yellow triangles with red lining indicate the samples that were considered outliers (>4SD) and excluded from further analyses. Subsequently the analysis was repeated in the combined cohort (remaining NBS controls and cases) and 1 outlier (>4SD) was excluded.

© 2023 de Groot EL et al. *JAMA Ophthalmology.*

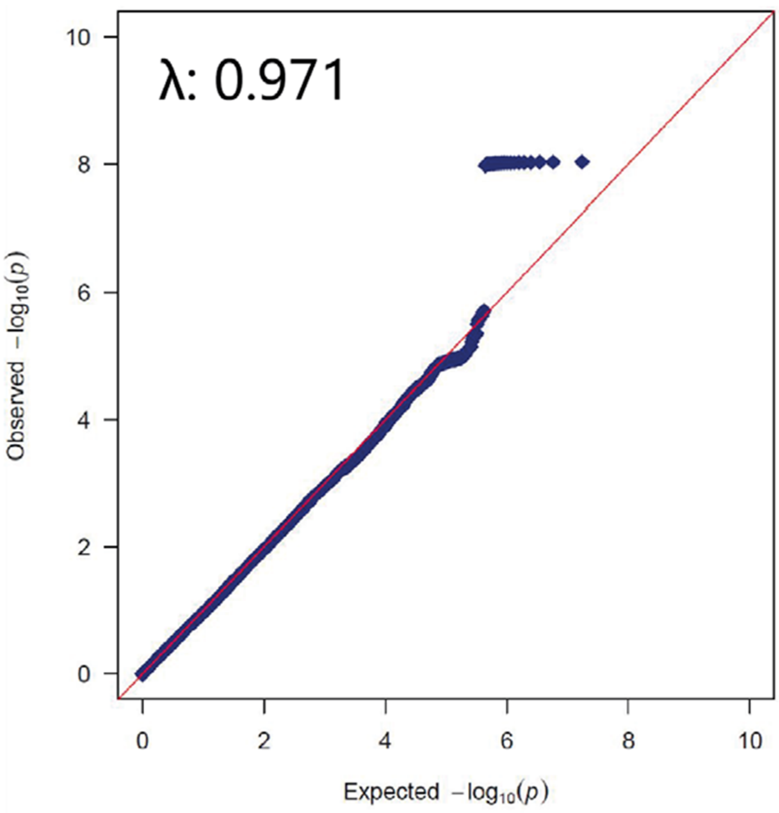

eFigure 2. Quantile-quantile plot. In a quantile-quantile plot every dot represents a single nucleotide polymorphism with its expected and observed p-value of the chi-square statistics. The associations were tested with a Scalable and Accurate Implementation of GEneralized mixed model including the covariates age, sex and the first 6 principal components. The red line indicates the normal distribution (lambda (λ) = 1.000). Because these SNPs are in near full linkage disequilibrium, they exhibit nearly identical association signals (*P*-value).

© 2023 de Groot EL et al. *JAMA Ophthalmology.*

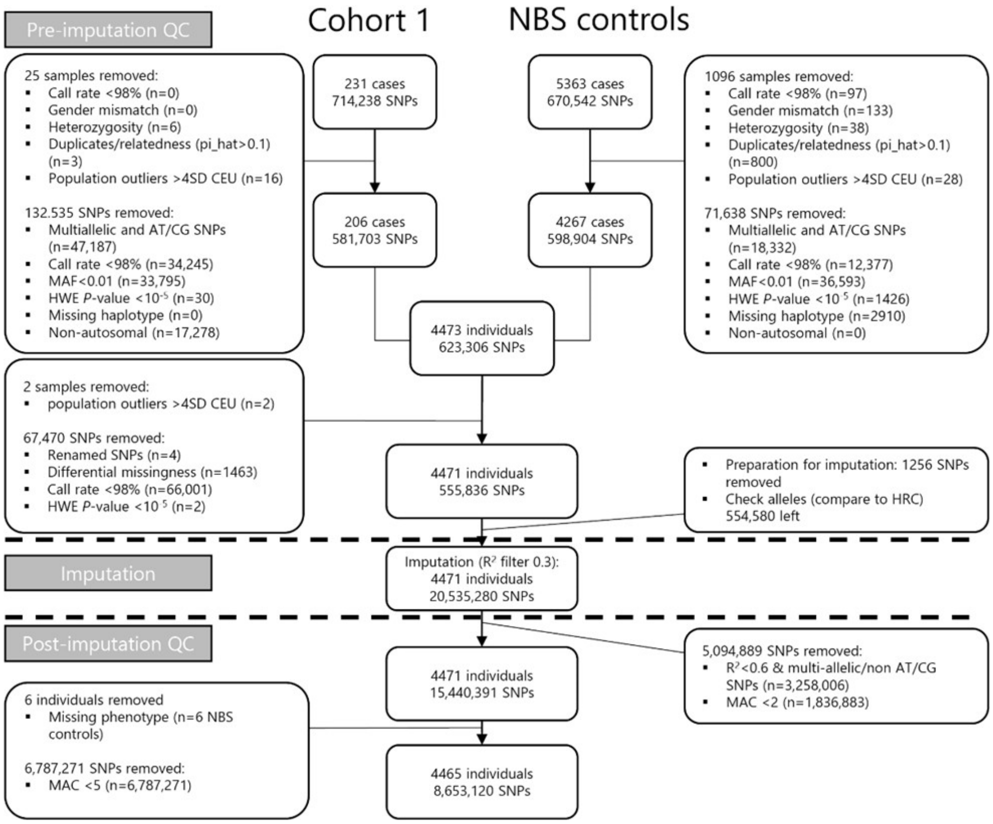

eFigure 3. Overview of the quality control steps. First, we performed quality control for both datasets separately (Cohort 1 and NBS controls). Next the datasets were merged and again subjected to stringent quality control. After imputation SNPs with an allele count of less than 5 were removed. In total 4465 cases and 8,653,120 SNPs were analyzed.

© 2023 de Groot EL et al. *JAMA Ophthalmology.*

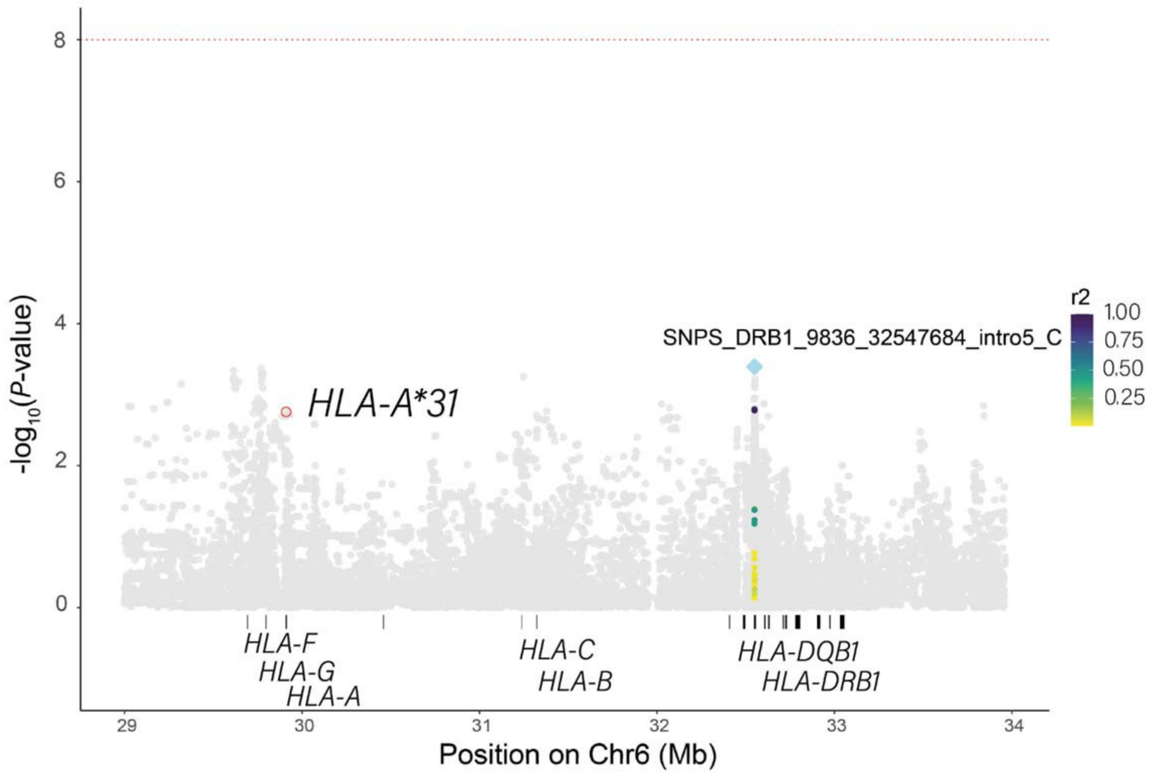

eFigure 4. Association results of the Major histocompatibility complex (*MHC*) region. This plot visualizes the association results of the *MHC* region on chromosome 6 after imputation. The x-axis represents the location on chromosome 6 including the annotation of the *HLA* genes and the y-axis represents the -$\log_{10}$ of the *P*-value. The associations were tested with a *Scalable and Accurate Implementation of GEneralized* (SAIGE) mixed model including the covariates age, gender and the first 6 principal components and association results are visualized as gray dots. The red dotted line indicates the threshold for genome-wide significant signals ($P < 5.0 \times 10^{-8}$). The most associated locus in the *MHC* region was a SNP at position 9836 of the gene body, genomic position 32547684 in intron 5 ($P = 3.7 \times 10^{-4}$) (blue triangle). Linkage disequilibrium (r2) between this SNP and SNPs in the region is shown in (see legend). Among the classical *HLA* alleles, *HLA-A*31* showed the lowest *P*-value (*P*=0.002) (red circle).

© 2023 de Groot EL et al. *JAMA Ophthalmology.*

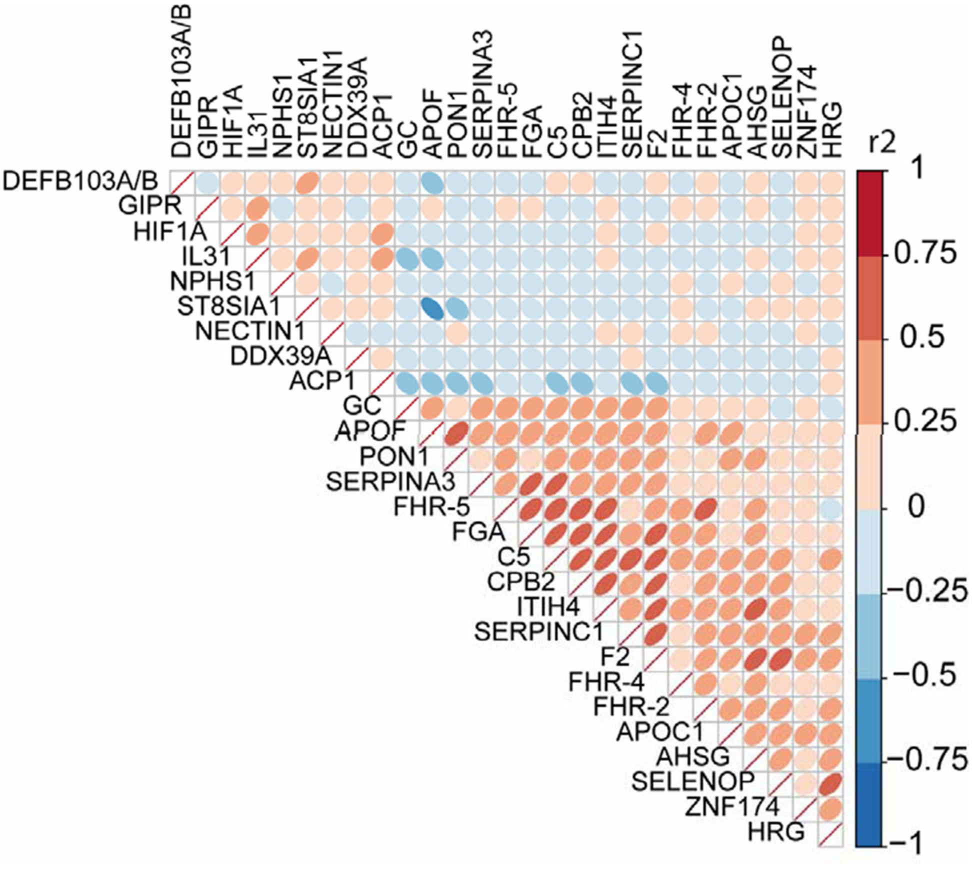

eFigure 5. Heatmap correlation. EDTA plasma was subjected to targeted 370-plex proteomics Olink array in 87 treatment naive patients with idiopathic multifocal choroiditis or punctate inner choroidopathy. This figure shows a heatmap of the correlation (color-coded from blue to red) between the 27 plasma proteins associated (likelihood ratio test P<0.05) with the genotype of rs7535263 in *CFH*.

© 2023 de Groot EL et al. *JAMA Ophthalmology.*

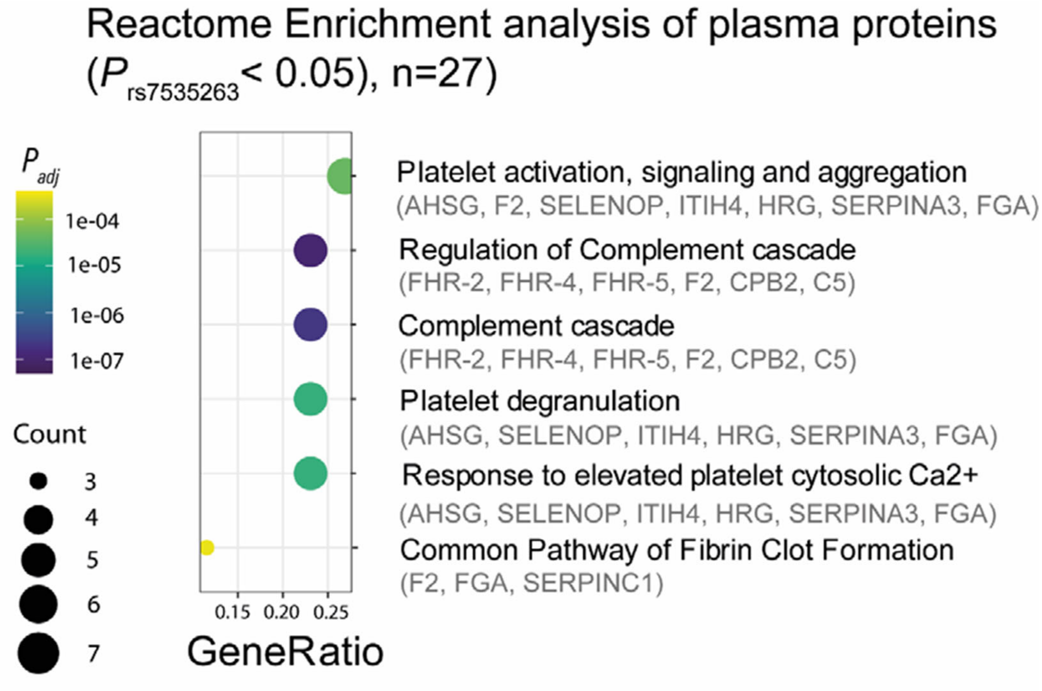

eFigure 6. Reactome enrichment analysis. The results of the reactome enrichment analysis of the 27 identified proteins (likelihood ratio test P<0.05) (eFigure 5). The top 6 pathways are shown and the adjusted P-value from enrichment analysis is color-coded. Plasma proteins that were annotated in each of the pathways are indicated in gray.

© 2023 de Groot EL et al. *JAMA Ophthalmology.*
