# Supplementary material for: Association of Risk Variants in the CFH Gene With Elevated Levels of Coagulation and Complement Factors in Idiopathic Multifocal Choroiditis
Source: JAMA Ophthalmol. 2023 Jul 6;141(8):737–45. doi: 10.1001/jamaophthalmol.2023.2557 (PMC10326733; doi:10.1001/jamaophthalmol.2023.2557)
Supplement: Supplement 3. — Data Sharing Statement [file jamaophthalmol-e232557-s003.pdf]

Data Sharing Statement

de Groot. Association of Risk Variants in the CFH Gene With Elevated Levels of Coagulation and Complement Factors in Idiopathic Multifocal Choroiditis. *JAMA Ophthalmol*. Published July 06, 2023. doi:10.1001/jamaophthalmol.2023.2557

Data
Data available: No

Additional Information
Explanation for why data not available: Summary statistics are available via DataverseNL (https://doi.org/10.34894/NXYU9W). Individual-level genotype data and associated personal data are protected under the General Data Protection regulation or GDPR. Please contact corresponding author for access to individual-level genotype data. Access requires the PI and an institution signing official to sign a Data Transfer Agreement (DTA). Supplementary contractual safeguards and provisions may be necessary for parties outside the EU/EEA.
